# Integrating intracellular nanovesicles into integrin trafficking pathways and beyond

**DOI:** 10.1007/s00018-022-04371-6

**Published:** 2022-06-03

**Authors:** Gabrielle Larocque, Stephen J. Royle

**Affiliations:** 1 The Francis Crick Institute, Midland Road, London, NW1 1AT UK; 2 Centre for Mechanochemical Cell Biology, Warwick Medical School, Gibbet Hill Road, Coventry, CV4 7AL UK

**Keywords:** Intracellular nanovesicles, Migration, Integrins, Membrane trafficking, TPD54, Rab30

## Abstract

Membrane traffic controls the movement of proteins and lipids from one cellular compartment to another using a system of transport vesicles. Intracellular nanovesicles (INVs) are a newly described class of transport vesicles. These vesicles are small, carry diverse cargo, and are involved in multiple trafficking steps including anterograde traffic and endosomal recycling. An example of a biological process that they control is cell migration and invasion, due to their role in integrin recycling. In this review, we describe what is known so far about these vesicles. We discuss how INVs may integrate into established membrane trafficking pathways using integrin recycling as an example. We speculate where in the cell INVs have the potential to operate and we identify key questions for future investigation.

## Introduction

Membrane trafficking is a fundamental process by which cellular cargoes are transported around the cell via lipid carriers. Whether it is for nutrient intake or receptor transport, lipid homeostasis, or growth factor secretion, this process is highly regulated. Not surprisingly, defects in such a crucial process can lead to catastrophic consequences such as Alzheimer’s disease, diabetes, and cancer [1]. Membrane trafficking systems are also widely used by pathogens such as viruses to infect their hosts [2]. It is therefore understandable that this field of cell biology has received so much attention over many years. The numerous membrane trafficking pathways have been expertly reviewed elsewhere, so we will only give a general overview here [3–6].

Consider the life cycle of a cell surface receptor. Transmembrane receptors are synthesized at the endoplasmic reticulum (ER) and need to be transported in membranous carriers toward the Golgi apparatus. In this compartment, their post-translational modification is finalized and they are sorted into the correct carriers toward the plasma membrane (PM). It is at the cell surface that they can function by binding their cognate ligand and initiating a cellular signaling event. The receptors, with or without their ligand, can then be internalized in a process called endocytosis and reach another major sorting organelle, the sorting endosome [3]. Once there, cargoes are sorted according to whether they need to be degraded (toward the late endosomes and lysosomes) or recycled (directly to the PM, toward the recycling endosomes or toward the Golgi apparatus) for more rounds of ligand binding [5]. Therefore, during the life of a receptor, it must travel in many different carriers, on several pathways, passing through numerous sorting stations.

The cytoplasm of a cell is a busy environment [7, 8]. Between the ER, the mitochondria and the actin, microtubule, and intermediate filament cytoskeletons, it seems that for a larger transport vesicle, finding its way can be a tough thing to do. This is probably also why larger trafficking organelles such as the lysosomes or Golgi apparatus are less mobile and serve as hubs for transport. Mobile carriers tend to be smaller: the PM- or Golgi-derived clathrin-coated vesicles (CCVs) are 50–100 nm in diameter, the ER-derived COPII-coated vesicles are 60–70 nm, and intra-Golgi transport vesicles 70–90 nm [9–11]. Until recently, the smallest known vesicular carriers were the neuronal synaptic vesicles (SVs, 33–42 nm, [12, 13]) or the ubiquitous intraluminal vesicles (ILVs) (20–100 nm, [14, 15]). Despite being heavily investigated, the field had not formally identified a new type of carrier in roughly 20 years. In 2020, a new class of transport vesicle was identified. These carriers, called intracellular nanovesicles (INVs), are very small (30 nm), yet carry a diverse set of cargoes, and operate on a variety of trafficking pathways. In this review, we will summarize the recent findings concerning INVs and explore their significance in various membrane trafficking pathways, more specifically in integrin trafficking and cell migration.

## Intracellular nanovesicles

### How were they found?

INVs were found by investigating the cellular role of Tumor protein D54 (TPD54, TPD52L2), a protein that is currently the best marker for these vesicles. TPD54 is a very abundant protein in HeLa cells (top 3% most abundant, [16, 17]). It is part of the Tumor protein D52-like protein (TPD52) family, along with TPD52, TPD53 (TPD52L1), and TPD55 (TPD52L3). The family members are ubiquitously expressed, apart from TPD55, which is restricted to testis [18].

**Fig. 1:**
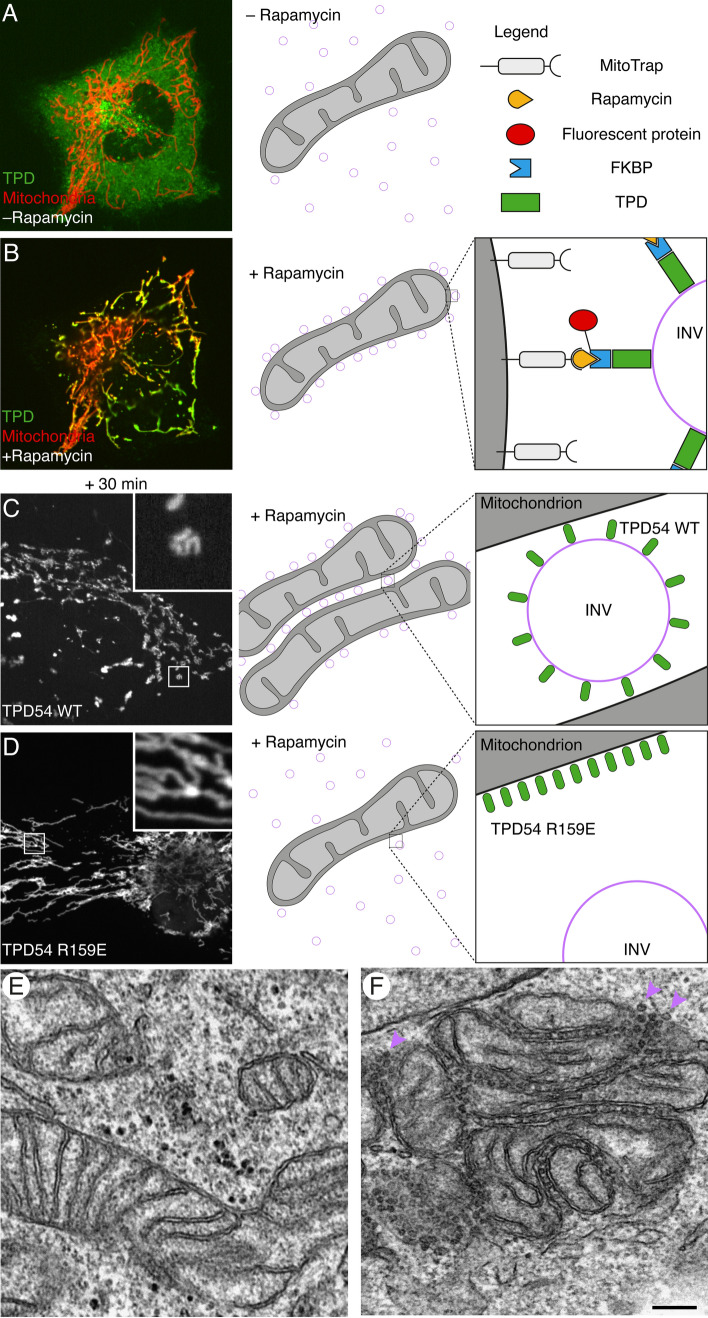
Discovery of INVs by relocalizing TPD54 to mitochondria. (**A–D**) TPD54 with an FKBP tag can be dimerized with an FRB-tagged protein on the mitochondria (MitoTrap) using rapamycin. Left panels show confocal micrographs of typical cells. Schematic diagrams (right) explain the result on the left, INVs are purple circles. No TPD54 relocalization (**A**) and shortly after relocalization (**B**). 30 min after TPD54 WT relocalization (**C**) or relocalization of a mutant, R159E, that cannot bind INVs (**D**). **E–F** Electron micrographs showing the mitochondria before (**E**) or 30 min after (**F**) TPD54 relocalization. Three INVs are highlighted with purple arrows. Scale bar, 200 nm. Micrographs © 2020 and 2021 Larocque et al. Originally published in *Journal of Cell Biology*. 10.1083/jcb.20181204410.1083/jcb.202009028

Knocksideways (KS) is a method to inhibit the function of a protein or to discover its binding partners (Fig. 1) [19, 20]. This technique is based on the binding of the prolyl isomerase FK506-binding protein 12 (FKBP12) to the mammalian target of rapamycin (mTOR) by the drug rapamycin. Usually, the FKBP domain of FKBP12 is added to the protein of interest, and the FKBP12-rapamycin-binding (FRB) domain of mTOR is on a fusion protein also containing the mitochondrial outer membrane import signal of Tom70p, called MitoTrap (Fig. 1A, B). The result is that the protein of interest is relocalized to the mitochondria. This inactivates the protein, since it is removed from its correct functional location; moreover, its binding partners may also be relocalized allowing for their identification [21]. For true KS, all of the protein needs to be relocalized, so the endogenous protein of interest is either depleted and a tagged version is expressed or the endogenous protein is directly tagged with FKBP [22].

To investigate the role and binding partners of TPD54, the protein was relocalized to mitochondria (Fig. 1B). Within minutes, the mitochondria aggregated, and when examined by electron microscopy, it was clear that the aggregation was caused by numerous, uniformly small vesicles gluing the mitochondria together [23]. So rather than removing a soluble pool of protein, the TPD54 associated with vesicles was moved to the mitochondria, together with the vesicles themselves. TPD54 binds the membrane of INVs (discussed below), and there are multiple copies of TPD54 on each INV; this is the reason why, when relocalized using this method, they glue the mitochondria together (Fig. 1C, E, F). These vesicles were named INVs and their presence in normal cells was confirmed by stochastic optical reconstruction microscopy (STORM) in HeLa cells using labeling of endogenous TPD54 [23].

### What are the molecular characteristics of INVS?

To determine the molecular components of INVs, three approaches have been used. First, relocalizing TPD54 to the mitochondria and quantifying the changes in the localization of any protein of interest. As described above, this method also causes aggregation of mitochondria which can also be used as a readout for the presence of the relocalized protein on INVs. Second, imaging INVs directly. Since the INVs are below the resolution limit of light microscopy (theoretical limit: 200 nm, INV size: 30 nm), they cannot be clearly seen. However, their presence can be measured due to their mobility, as the spatiotemporal variance of a fluorescently tagged protein on an INV. Third, proteomic approaches can be used to identify proteins in INVs following crude purification via TPD54 [23, 24].

#### TPD52-like proteins

A combination of all three approaches revealed that two other TPD52-like proteins, TPD52 and TPD53, are also present on INVs [24]. TPD52-like proteins are able to form homo- and heterodimers via their coiled-coil domain, but their ability to bind INVs is independent of dimerization [24–26]. TPD52-like proteins are *~* 50% similar (*~* 40% identical) with regions of high conservation in their C-terminal regions, suggesting that this region may be responsible for targeting to INVs and that a similar mechanism operates for all members of the family. Two studies have examined how TPD54 binds INVs. Reynaud et al. [27] found that TPD54 has sequences corresponding to four amphipathic helices (AH1-4) downstream of the coiled-coil region. The third helix (AH3) corresponds to an amphipathic lipid packing sensor (ALPS) motif which is known to govern membrane binding in a curvature-dependent manner [28]. Overall, this downstream region is disordered; however, when TPD54 protein is incubated with liposomes, TPD54 becomes ordered upon binding to the membrane. Both studies showed that TPD54 has a preference for high curvature membranes in vitro [24, 27]. The ALPS-containing AH3 is crucial for binding [27]. However, this preference only partially explains the targeting of INVs in cells. Mutation of hydrophobic residues in AH2 and AH3 but not AH1 or AH4 caused mislocalization of TPD54 in cells, suggesting that AH3 is not the only determinant for INV-binding [27]. Moreover, mutation of charged residues in AH1, AH3, and AH4 all caused mislocalization in cells, but none of these affected binding to high curvature vesicles in vitro [24]. This suggests that other factor(s) are involved in TPD54 binding to INVs. Precisely how TPD52-like proteins are targeted to INVs in cells requires further investigation.

The association of TPD54 with INVs is strong with almost all of the TPD54 in cells being associated with vesicles, according to fluorescence recovery after photobleaching experiments [23]. For this reason, TPD52-like proteins are the current best markers for INVs. An important thing to note is that they are not inert markers, since there is evidence that TPD54 is needed for the function of the INVs. Indeed, a TPD54 mutant (Fig. 1D) that is not associated with INVs fails to rescue a migration phenotype caused by a TPD54 depletion [24]. This set of experiments will be covered in the section below.

#### Rab GTPases

There are over 60 different Rabs now identified in mammalian cells, and each one regulates a pathway of membrane trafficking [29]. They switch between an active and inactive state, depending on whether they are bound to GTP or GDP, respectively. This allows for a wide range of proteins to bind preferentially to either form, and these binding partners dictate the many roles that Rabs have; for example vesicle fusion, lipid composition, or cytoskeletal transport [30]. By having its own Rab, a transport vesicle can select the right cargo, present the right lipid, ride the right motor, and fuse with the right membrane. The INVs are no exception, and they appear to be associated with at least 15 Rabs from a wide range of trafficking pathways [23]. These include the anterograde, intra-Golgi, and endosomal recycling pathways. The current model is that each INV has one specific Rab, but collectively INVs represent a cross-section of trafficking pathways in the cell and are therefore associated with many Rabs.

The Rab complement of the INVs was identified using the co-relocalization approach described above. TPD52, TPD53, or TPD54 was relocalized to mitochondria in HeLa cells co-expressing 43 different Rabs tagged with GFP. Some Rabs co-relocalized with all three TPD52-like proteins (Rab1a, 1b, 14, 26 and 30), some co-relocalized with both TPD52 and TPD54 (Rab11a) or TPD53 and TPD54 (Rab12 and 43), and some only relocalized with one TPD (54: Rab3a, 4a and 25; 53: 33b and 19b; 52: Rab10 and 17) [23, 24]. Understanding the pathways, these Rabs are on might give us insights into where the INVs are, and this will be discussed in the section below.

#### SNAREs

Soluble N-ethylmaleimide-sensitive factor attachment protein receptors (SNAREs) are required for the fusion of an incoming vesicle with the right acceptor membrane [31]. SNAREs are divided into two categories: R- or Q-SNARE, based on which amino acid is present in the ionic layer (R- arginine, Q- glutamine). For membrane fusion, four helices need to come into contact: one helix from the R-SNARE, one helix from the Qa-SNARE, and two helices from a third Qbc-SNARE [32]. Usually, Q-SNAREs are also classified as t-SNAREs (t, target) and R-SNAREs are v-SNAREs (v, vesicle), but some exceptions exist [33]. Of the 38 SNAREs, ten were put in the co-relocalization assay with TPD54: five Q-SNAREs (syntaxin (Syt) 6,7,8,10 and 16), and five R-SNAREs (vesicle-associated membrane protein (VAMP) 2,3,4,7 and 8). Four R-SNAREs co-relocalized (VAMP2, 3, 7, and 8), whereas none of the Q-SNAREs did [23].

VAMP2 (also known as synaptobrevin-2) is mainly found in neuronal cells and is necessary for synaptic vesicle exocytosis via binding with synaptosomal-associated protein, 25 kDa (SNAP25), and synaptotagmin-1 [34]. VAMP2 governs other exocytic events in other cell types; for example, glucose transporter type 4 (GLUT4) delivery in adipocytes [35]. VAMP3 acts on different trafficking pathways. It allows the transport of cargoes to the plasma membrane, such as the Weibel–Palade bodies (WPB) in endothelial cells [36], and it plays a role in extracellular matrix (ECM) remodeling by delivering the matrix metalloproteinase MT1-MMP from the sorting endosome to the PM [37]. It is also involved in pathways such as the sorting endosome-to-Golgi transport [38], cilium formation [39], in fibrinogen endocytosis in platelets [40], and recycling endosome-to-autophagosome fusion in response to bacterial infection [41]. VAMP7 is also found on many pathways, from endocytosis, to endosome maturation, cilium formation, autophagy, and exocytosis [42, 43]. Finally, VAMP8 is needed for fusion between the autophagosomes and lysosomes [44], and fusion with the plasma membrane of different types of transport structures, such as exosome released from MVBs [45], WPB [46], mucin exocytosis from goblet cells [47], or recycling endosomes [48]. Therefore, like the Rab complement of INVs, the variety of SNAREs found on INVs points to these vesicles being involved in a range of trafficking pathways. Potentially, INVs may also be involved in some of the “specialized” trafficking pathways involving these SNAREs in specialized cells.

### Which trafficking pathways?

The membrane trafficking pathways on which the INVs work are known from two sets of experiments. The first set comes from interfering with the expression of TPD54, and the second from the array of Rab GTPases co-relocalized with the INVs. Depleting TPD54 has been shown to impair two main pathways: the anterograde biosynthetic pathway and the endosomal recycling pathway. Indeed, using the retention using selective hooks assay (RUSH, [49]), TPD54-depleted cells showed significantly slower ER to plasma membrane trafficking and intra-Golgi transit. It was also shown that a TPD54 knockout or knockdown causes disruption in Golgi organization, confirming its role in Golgi trafficking. TPD54 depletion also causes significant delays in integrin and TfR recycling, but not endocytosis.

**Fig. 2:**
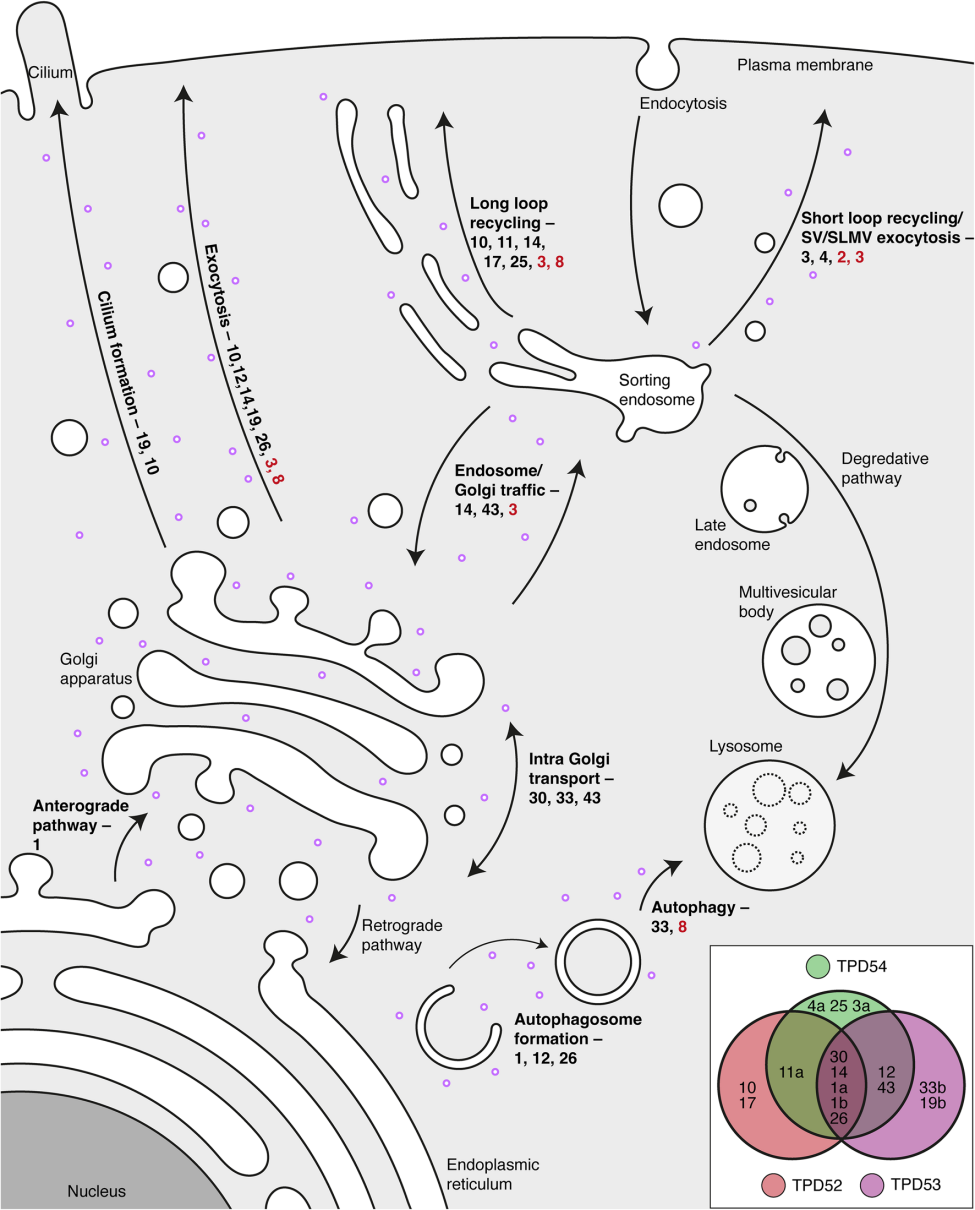
INVs participate in multiple trafficking pathways. Schematic representation of the membrane trafficking pathways (bold) regulated by the INVs (purple circles) as shown by Rabs (black number) or SNAREs (red number) that co-relocalize with TPD54, or a TPD54 depletion. Bottom right: Euler plot to show the Rabs (numbers) identified in the relocalization screens and how they are linked to TPD52-like proteins

Rabs are well-established markers of all membrane trafficking pathways, where they oversee multiple steps [29]. The set of Rabs co-relocalized with the INVs is therefore indicative of which pathways the INVs are on, and confirmed some of the pathways identified by the phenotypes caused by TPD54 depletion (Fig. 2). They map to the biosynthetic anterograde pathway (Rab1, Rab30, Rab14, Rab26, Rab12, Rab3, Rab10, Rab19), intra-Golgi traffic (Rab30, Rab43, Rab33, Rab19), and endosomal recycling pathways (Rab14, Rab11, Rab4, Rab25, Rab17). Some Rabs identified to be associated with INVs are on other pathways and give insights into alternative pathways where INVs might function.

#### Rab30

Rab30 is the Rab that co-relocalized most extensively with all three members of the TPD52-like protein family and deserves special interest, since it is not well characterized. Its cellular localization and appearance on subresolution structures are particularly reminiscent of the TPD52-like proteins. It has been implicated in the maintenance of the Golgi apparatus integrity without having a role in anterograde or retrograde transport [50]; however, this has recently been questioned [51]. An argument in favor of Rab30 having a role in anterograde and retrograde transport from the Golgi comes from a screen to identify Rab effectors in *Drosophila*. The authors found that some of these effectors are subunits of the Golgi-associated retrograde protein (GARP) complex (involved in endosome-to-Golgi vesicle tethering), and the exocyst complex (tethering of vesicles going from the Golgi apparatus to the plasma membrane)[52]. Another interesting Rab30 effector is GC5168, the fly homolog of WDFY1/2 [52]. WDFY2 binds to VAMP3 (which is found on INVs) and membranes rich in PI(3)P on endosomal tubules and regulates the secretion of the matrix metalloproteinase MT1-MMP [37]. It is possible that INVs have a role in this process if it contributes to their role in cell migration. Finally, Rab30 also binds to the phosphatidylinositol 4-kinase PI4KB, at least in the context of a bacterial infection [53]. This is needed for the production of PI(4)P on group A streptococcus-containing autophagosomes-like vacuoles (GcAVs), a process that results in the degradation of the bacteria [53]. Whether Rab30 produces PI(4)P on INVs is an interesting open question.

#### Biosynthetic anterograde pathway

The biosynthetic anterograde pathway takes newly synthesized cargo from the ER to the Golgi, through the Golgi and out to the plasma membrane or to endosomes (Fig. 2). Rab1 is a well-established regulator of the early secretory pathway controlling traffic between the ER and the Golgi [54]. Some of its effectors are the Golgi-resident vesicle tethers GM130, Golgin-84, and giantin, allowing the fusion of Rab1-positive vesicles with the Golgi apparatus and maintaining its integrity [55–58]. Rab14 has also been observed on this pathway, regulating the transport from the TGN to the plasma membrane [59]. Rab12 is localized to the Golgi apparatus and early reports suggest a role in secretion [60, 61]. Rab10 was first identified around the Golgi apparatus and has been involved in the polarized trafficking of cargoes from the basolateral regions to common endosomes in epithelial cells [62, 63]. Rab3 is an exocytic Rab mainly associated with SVs, but also with insulin or pancreatic zymogen granules, and many others [64]. The protein has 4 isoforms (Rab3a, b, c, and d) which are mostly redundant [65]. In neurons, the synaptic vesicle-bound Rab3, together with Rab3-interacting molecule (RIM), Rabphilin, and MUNC13, plays a role in vesicle docking priming and fusion [66]. Finally, Rab26 has been involved in Golgi to plasma membrane transport of the alpha2-adrenergic receptor [67], and Rab11 has been found on the TGN and regulates the secretory pathway [68].

The Golgi apparatus is formed by a stack of membranes arranged in cisternae. There is a constant arrival and departure of membrane carriers to and from the Golgi, so preserving the architecture of the Golgi is crucial for its sorting role and the correct localization of the many proteins involved in this sorting. It seems that INVs modulate this process, as it was also shown that a TPD54 knockout or knockdown causes disruption in Golgi organization. Moreover, Rab43 is required for the retrograde transport of Shiga toxin from the sorting endosomes/recycling endosomes to the Golgi [69], Rab14 was seen on the retrograde EE-to-Golgi pathway, transporting cargoes such as GLUT4 [70, 71], and Rab33 localizes to the Golgi medial cisternae and regulates Golgi–ER retrograde transport in a pathway that also involves Rab6 [72, 73]. These three Rabs regulate the integrity of the Golgi by modulating what comes in and what goes out.

#### Endocytic recycling

INVs participate in the recycling of cargo back to the plasma membrane, but are not involved in endocytosis. TPD54 depletion had no effect on the internalization of integrins or TfR, and none of the endocytic Rabs (Rab5, Rab21, or Rab22) co-relocalized with any TPD52-like protein [23, 24]. Moreover, transmembrane cargo with a dileucine motif was found in INVs, but does not appear there until 60 min after endocytosis. There is however data, suggesting that the INVs affect the endocytic recycling (Fig. 2). Recycling of integrins and TfR were significantly affected by depletion of TPD54 and many of the Rabs co-relocalized with INVs also suggest a recycling role. For example, Rab14 regulates the endocytic recycling of a disintegrin and metalloproteinase 10 (ADAM10), a pathway involved in cadherin removal from cell–cell junctions. Indeed, ADAM10 is selectively sorted in carriers going to the cell surface in an intermediate compartment between the Rab4/Rab5-positive early endosome and the Rab11-positive recycling endosomes [74]. Rab17 is specific to epithelial cells, where it regulates the transport between the basolateral plasma membrane and the apical domain and apical recycling [75, 76]. Rab10 is involved in the formation of tubular endosomes with KIF13, an organelle responsible for the recycling of cargoes internalized via clathrin-independent endocytosis [77].

The main evidence for a recycling role comes from the co-relocalization of the main recycling Rabs: Rab11, Rab4, and Rab25. Rab11 has three isoforms, Rab11a, b, and c. Rab11c is also known as Rab25, whereas Rab11 is used for isoforms a and b. Rab11 is found in sorting endosomes, in a distinct domain to that Rab5 and Rab4 [78]. Cargoes in this Rab11-positive domain are transported via a "long loop" toward the recycling endosomes, whereas cargoes going back to the plasma membrane directly via the Rab4-positive domain use the "short loop" [79]. The expression of Rab25 is restricted to epithelial cells, where it localizes to the sub-apical region and colocalizes with Rab11a [80]. This Rab has been identified as being a tumor promoter or suppressor, a role that might be dictated by the presence or absence of its effector, Rab coupling protein (RCP) [81]. In a breast cancer cell line, Rab25 has been shown to bind integrin α5β1 in a GTP-dependent manner, whereas the closely related Rab11a does not. This binding influences cellular migration and invasiveness in two ways. At the cell front in pseudopodia, Rab25 keeps a pool of inactive integrins (ECM-free) by recycling the dimer to the PM after internalization [82]. This allows the pseudopodia to make new adhesions and promotes migration. Active integrins α5β1 (fibronectin-bound) are ubiquitinylated and degraded in the lysosomes [83], unless CLIC3 is expressed. It has been shown that active integrins are transported in a retrograde manner to CLIC3-positive late endosomes/lysosomes, where CLIC3 sorts the integrins back to the PM at the back of the migrating cell, to avoid degradation [84]. Like Rab25, TPD54 is overexpressed in certain types of cancer and its expression is linked to a more aggressive metastatic phenotype [81, 85]. Also, like Rab25, TPD54 is involved in integrin trafficking, cell migration, and invasion [24].

Finally, Rab4 is another well-characterized Rab. As mentioned above, it is found in the sorting endosome in a distinct domain to that of Rab5 and Rab11, and is not found in recycling endosomes [78]. Unlike Rab11, however, it stays in the sorting endosomes, where it regulates the recycling of a number of cargoes, including GLUT4, integrins, and TfRs [86, 87]. Another interesting role of Rab4 is the regulation of the formation of synaptic-like microvesicles (SLMVs) [88]. SLMVs are a type of vesicles present in neuroendocrine cells that resemble the neuronal SVs in morphology, cargo, and release mechanism [89–91]. SLMVs are formed from two distinct locations, the PM and from tubules on sorting endosomes, in a Rab4-dependent manner [88, 92]. The SLMVs then need to be transported toward the Golgi apparatus and gain Rab3 for subsequent fusion with the PM [93]. SVs, SLMVs, and INVs are similar in size, and TPD52, TPD53, and TPD54 are ubiquitously expressed. It is therefore conceivable that protocols aiming at isolating SVs and SLMVs might also isolate INVs, and that proteomic analysis may include an INV component. Indeed, the TPDs are found in some of these datasets [94, 95]. Another possibility is that these vesicle types are not distinct; just different flavors of the same vesicle class. Interestingly, exogenously expressed synapsin and synaptophysin in fibroblasts can cluster small synaptic-like vesicles from the endosomal pathway [96, 97]. The authors do not speculate on the nature of these synaptic-like vesicles, but their nature is strikingly similar to INVs. These data raise the question of whether SLMVs and SVs are a subtype of INVs.

#### Cilium formation

The involvement of INVs in this pathway has not yet been examined, but two of the Rabs co-relocalizing with INVs are associated with cilium formation (Fig. 2). Rab10 was shown to be involved in ciliogenesis, where it interacts with the exocyst complex at the cilium base and modulates membrane transport [98, 99]. Rab19 was mostly uncharacterized until very recently. As a close homolog to Rab43, it was found at the Golgi apparatus in *Drosophila*, and interacted with Pollux, an ortholog of TBC1D1 and TBC1D4/AS160, which both regulate GLUT4 trafficking [52, 100]. Still, in *Drosophila*, Rab19 was seen colocalized with Huntingtin-positive vesicles, suggesting that it might have a role in axonal transport [101]. Very recently, the role of Rab19 in mammalian cells was investigated. Here, it was reported that Rab19 is involved in ciliogenesis by recruiting the HOPS tethering complex to the pericentriolar region, promoting clearance of the actin cortex that is needed for cilium membrane and microtubule extension, a process regulated by TBC1D4, a Rab19 GAP [102].

#### Autophagy

Autophagy (self-eating) is a process during which the cell degrades different components in response to stress or nutrient deprivation, and recycles these components for reuse (Fig. 2). It is also a way to clear potentially harmful material, such as broken mitochondria or pathogens [103]. Membranes from different origins are brought to the material needing to be degraded until this phagophore can be closed by the ESCRT machinery. The resulting autophagosome fuses with lysosomes and its content is degraded [104]. This process is regulated by the autophagy-related proteins ATGs and many Rabs are also involved [105]. For example, PI(3)P are lipids needed for autophagosome production, and Rab1 recruits and activates the PI(3)P generating protein complex VPS34/VPS15/Beclin/ATG14L [106, 107]. It also regulates the localization of myotubularin-related protein 6 (MTMR6), a lipid phosphatase making PI(3)P from PI(3,5)P_2_. Together, they alter the formation of the omegasome, a step in autophagosome formation [108]. As mentioned above, Rab12 regulates EE–Golgi transport, but it has mainly been shown to be involved in autophagy, a role that is regulated by the ULK-dependent phosphorylation of the Rab12 GEF, DENND3 [109, 110]. In the case of Rab11, it binds to TBC1D14/ULK1, where at least a population of recycling endosomes are added to the forming autophagosomes [111]. Rab26 has a similar function to its effector ATG16L1, where they redirect synaptic vesicles to autophagosomes [112]. Finally, Rab33 and Atg16L1 are involved in autophagosome fusion with lysosomes [113].

Given the involvement of these Rabs which are associated with INVs, how could INVs fit into the autophagy pathway? The phagophore, the organelle that will become the autophagosome, is supplied with membrane by Atg9 vesicles [114]. The size of these Atg9 vesicles is similar to that of INVs: 30–60 nm [115, 116]. Moreover, TPD54 was among the top hits in a proteomic analysis of ATG9A-positive structures [117]. It is possible that Atg9 vesicles might be a subtype of INVs, and that INVs, alongside the Rabs associated with them, regulate autophagy. Alternatively, Atg9 vesicles have recently been shown to play a role in integrin transport and cell migration, and so the link between these vesicle types may occur in a non-autophagy role [118].

In this section, we saw that Rabs associated with INVs highlight the same pathways identified by functional assays: the biosynthetic anterograde pathway (Rab1, Rab30, Rab14, Rab26, Rab12, Rab3, Rab10, Rab19), intra-Golgi traffic (Rab30, Rab43, Rab33, Rab19) and endosomal recycling pathways (Rab14, Rab11, Rab4, Rab25, Rab17). Whether the other pathways marked by other Rabs associated with INVs (autophagy, endocytic retrograde transport, ciliogenesis) are also regulated by the INVs is an interesting open question. For easy reference, this information is summarized in Table 1.

**Table 1:** Reported localization of the Rab GTPases and SNAREs that co-relocalized with the TPDs

Organelle	Rab GTPase	SNARE	TPD present
Golgi apparatus	1, 3, 10, 11, 12, 14, 26, 30, 33, 43	VAMP2, VAMP3, VAMP7, VAMP8	Yes
Sorting endosome	4, 11, 14, 43	VAMP3, VAMP7	Yes
Recycling endosome/endocytic recycling compartment	10, 11, 17, 25	VAMP3, VAMP8	Yes
Autophagosome	1, 11, 12, 26, 33	VAMP3, VAMP7, VAMP8	Present in mass spectrometry dataset
Primary cilium	10, 19	VAMP3, VAMP7	?
Synaptic vesicle/synaptic-like microvesicle	3, 4	VAMP2	Present in mass spectrometry dataset

### What do INVs transport?

Given the variety of pathways that INVs are involved with, it is likely that a wide variety of cargo will transit via INVs. Currently, there is no proteome of INVs available to answer this definitively; however, we do have some information about the cargo that INVs transport. Using the relocalization assay, it was found that cargoes with a dileucine endocytic motif ([D/E]XXXL[L/I/M]) are preferred over cargoes with a tyrosine motif ([F/Y]XNPX[Y/F] or YXXϕ; X is any amino acid and ϕ is a bulky hydrophobic amino acid) [23]. The cation-independent mannose-6-phosphate receptor (CIMPR), which contains several different endocytic motifs, was also found in INVs. Most recently, when investigating the role of TPD54 in cell migration, it was found using a recycling assay, immunoprecipitation, and proximity labeling that INVs transport several different integrin heterodimers. This finding is interesting, because the integrins are large molecules that bind the extracellular matrix. For example, αIIbβ3 integrin is 11 nm long in its extended conformation [119]. This suggests that although INVs are 30 nm in diameter, they are able to carry sizeable cargoes around. INV cargoes CIMPR (26 × 13 nm) and TfR (8 × 10 nm) are also quite large [120, 121]. Whether INVs do this by adapting their size or by limiting the number of large cargo molecules they carry is an interesting question. If INVs limit cargo transport, then it is possible that this could be a mechanism for precisely controlling the amounts of cargo flowing through a pathway.

### INVs in cell migration and integrin trafficking

The transport of integrins in INVs is interesting because of the central role that integrin trafficking has in cell migration and cancer. The expression of INV markers TPD52 and TPD54 is altered in different cancers [24, 85, 122–124], suggesting that the mechanism by which the INVs contribute to carcinogenesis may be via their role in integrin trafficking.

**Fig. 3:**
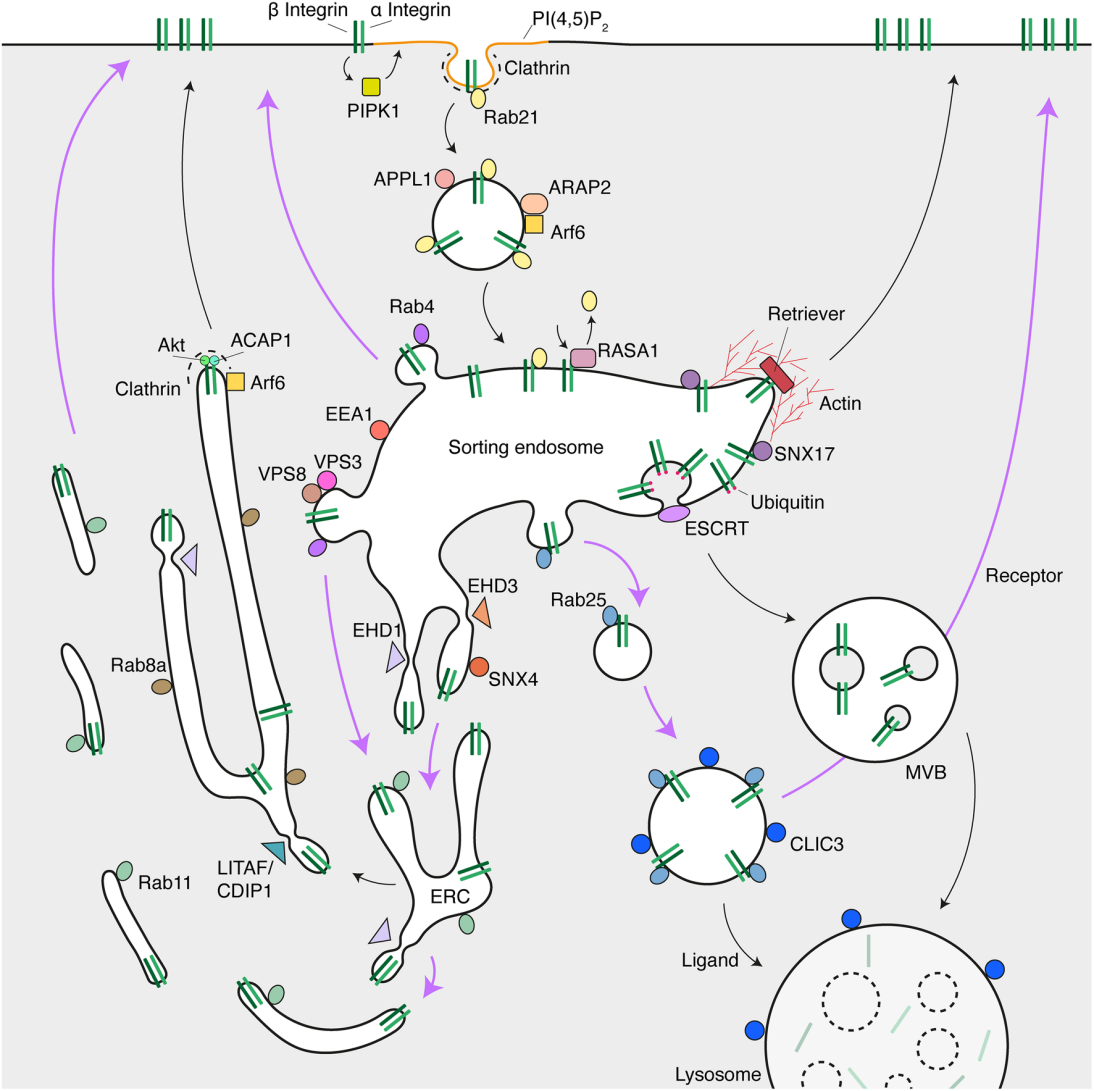
Integrin trafficking pathways. Schematic representation of the integrin trafficking pathways and their key players. Purple arrows indicate where INVs are likely to have a role

The integrins are cell surface receptors for different components of the ECM, such as fibronectin, vitronectin, or laminin. They ensure a mechanical connection with these components at sites known as adhesions. There are 18 α-subunits and 8 β-subunits which assemble in 24 possible heterodimers [125]. The formation and removal of cellular adhesions is a dynamic process that is required for the cell to move. Because integrins are transmembrane proteins, they need to be endocytosed and recycled during the removal and formation of adhesions. Integrins can also be degraded, but this fate is not favored as their half-life is 12–24 h [83, 84]. Precisely how integrins are trafficked depends on many things: heterodimer type and activation state, cell type, disease context, whether the integrin is on the basal versus apical region, and so on. This complex process has been the focus of many studies (expertly reviewed in [126–128]). We will present here a general overview of the different pathways involved in integrin traffic (Fig. 3).

### A brief overview of integrin trafficking

#### Internalization

The best-studied mode of integrin internalization is clathrin-mediated endocytosis (CME), although other modes of internalization have also been reported [129, 130]. The binding of the integrin dimer to the ECM stimulates the production of PI(4,5)P_2_ from PI(4)P by type I phosphatidylinositol phosphate kinase β (PIPKIβ) [131]. This recruits the endocytic machinery. The clathrin adaptor AP2, but also Dab2, ARH, Eps8, and Numb have all been shown to regulate integrin endocytosis. They have the ability to bind the NPxY/NxxY motif in the cytoplasmic tail of β integrins [128, 132]. Internalization of the integrin heterodimer is regulated by the endocytic Rab5 and Rab21, where Rab21 binds directly to the α subunit and Rab5 associates with the β subunit [133]. At least in the case of integrin α5β1, it was also shown that the Arf6 GAP ARAP2 (Arf GAP, Rho GAP, Ank repeat, and PH domain 2) mediates the transport of the dimer to phosphotyrosine-binding domain, and leucine zipper motifs 1 (APPL1)-positive pre-sorting endosomes, and that depletion of ARAP2 slows α5β1 internalization [134]. The internalized integrins are then transported toward the sorting endosome (Fig. 3).

#### Sorting

Once in the sorting endosome, RASA1 (p120RasGAP) competes with Rab21 for the same binding site on the α subunit. This allows the integrins to be recycled back to the cell surface. Indeed, silencing of RASA1 leads to an accumulation of integrins in the sorting endosome [135]. It has been shown that endosomal integrins are signaling platforms, where the focal adhesion kinase (FAK) is recruited and leads to suppression of anoikis (programmed cell death caused by adherent cell detachment) and metastasis [136].

From here, integrins can be sorted into different recycling pathways. Certain pathways recycle integrins directly from the sorting endosomes, while some first send them toward the juxtanuclear endocytic recycling compartment (ERC).

#### Recycling from the sorting endosome

The pathways involved in the direct recycling of integrins from the sorting endosomes are Rab4- or retriever-dependent [87, 137]. As seen earlier, Rab4 regulates the "fast" recycling of a number of cargoes. In migrating cells, the Rab4-dependent recycling of integrins is also growth factor-dependent. Indeed, in the absence of growth factors, integrins instead take the longer Rab11-dependent route [87]. Growth factors such as PDGF (platelet-derived growth factor) promote the phosphorylation of the kinase PKD1, which enables the binding of PKD1 to the cytoplasmic tail of β3 integrin [138, 139]. PKD1 phosphorylates the Rab5 effector Rabaptin-5, which then binds preferentially to Rab4 over Rab5, and promotes integrin recycling [140].

The retriever pathway has recently been characterized and is an alternative route away from degradation, since depletion of key components sends the integrins to the lysosomal pathway [141]. The retriever complex is similar to retromer and is formed of three subunits: DSCR3 (VPS26C), C16orf62 (VPS35L), and VPS29. It mediates the recycling to the plasma membrane of SNX17-bound cargoes, such as integrin β1, where SNX17 binds its NPXY motif [137]. It is recruited to the sorting endosomes by the addition of PI(3)P in the membrane by the lipid kinase VPS34 [142, 143]. PI(3)P also recruits another lipid kinase, PIKfyve, which makes PI(3,5)P_2_ from PI(3)P [144]. PI(3,5)P_2_ serves as a platform to then recruit other components, such as the Arp2/3 activator Wiskott–Aldrich syndrome protein and SCAR homologue (WASH) and CCDC22, CCDC93, and COMMD (CCC) complexes, which also bind to each other [137, 142, 145]. Retriever can then indirectly bind the integrins and regulate their recycling by binding to SNX17, CCC, and WASH [137, 142]. WASH activates the actin nucleator Arp2/3 and the F-actin generated promotes cargo trafficking and vesicle scission [146, 147]. The C-terminal Eps15 homology domain 1 protein (EHD1), recruited by SNX17, also regulates the scission of recycling vesicles [148]. A role for Golgi-localized gamma-ear containing Arf-binding protein-3 (GGA3) has been found in the recycling of integrins in association with SNX17. It was shown that GGA3 can regulate the endosomal localization of SNX17 and through interaction with Arf6, prevent integrins from accessing the degradative pathway [149] (Fig. 3). However, it remains to be clarified if the regulation of SNX17 by GGA3 affects the function of the retriever.

A recent study has shown that integrins, but not the TfR, can be transported to the ERC via Rab4-positive endosomes. CORVET (class C core vacuole/endosome tethering) participates in the fusion of Rab5-positive sorting endosomes. It shares four core subunits (VPS11, 16, 18, and 33a) with the late endosome tethering complex HOPS (homotypic fusion and protein sorting) and has two specific subunits, VPS3 and VPS8 [150]. It has been shown that VPS3 and VPS8 are needed for the delivery of integrins from the sorting endosome via Rab4-positive carriers toward Rab11-positive recycling endosomes before being sent to the plasma membrane [151].

#### Recycling from the ERC

Like many other cargoes, the integrins can also be sent to the ERC and rejoin the PM via the "slow" recycling pathway. The ERC is characterized by the presence of molecular markers, such as Rab11, EHD1, and Arf6. Cargoes in this compartment return to the plasma membrane via Rab11-positive carriers (CME and clathrin-independent cargoes) or longer, EHD1- and Rab8-positive tubular recycling endosomes (clathrin-independent cargoes) [152]. The ERC is regulated by a few different classes of molecules, the main ones being small GTPases, sorting nexins, and the EHDs (Eps15 homology domain). By binding to cargoes, some sorting nexins prevent cargo degradation by sorting them into recycling compartments [152]. We have seen earlier how SNX17 sorts integrins to the retriever-dependent pathway. SNX4 is responsible for sorting cargoes from the sorting endosome to the ERC. It does so with KIBRA by binding to the minus-end microtubule motor dynein [153], and/or the microtubule-binding protein α-taxilin [154]. SNX4 has been shown to be important for the traffic of the TfR and BACE1 [153, 155]. SNX4 and Rab11 both interact with reggie-1 (also known as flotillin-2) on the ERC, and this does indeed regulate the recycling of integrins [156, 157].

Other motors have been shown to be important for sorting into the ERC. Kinesin II is bound by the Rab11 effector Rab11-FIP5 (Rip11) and contributes to TfR recycling [158]. Since Rab11 is a well-established regulator of integrin trafficking, it is likely that the kinesin II/Rab11-FIP5 complex also participates in integrin recycling. Another Rab11 effector, Rab11-FIP2, acts as a scaffold between Rab11-positive carriers and the actin motor myosin 5b (myo5b or myoVb) [159, 160]. Similarly, the tubular endosomal Rab8a can also bind Myo5b [161]. Integrins are a Rab11 and Rab8 cargo, it is therefore likely that Myo5b participates in integrin recycling either via the Rab8 tubular endosome or the Rab11 recycling endosome [162] (Fig. 3).

The tubular endosomes are characterized by the presence of the membrane vesiculators EHD1 and EHD3 [163], and EHD1 has been involved in the recycling of integrins [164]. This role is orchestrated by the cytoskeleton organizing protein MICAL-L1 and Rab8a [165]. Recently, another set of proteins has been shown to participate in the scission of integrin-containing carriers from the tubular recycling compartment. Indeed, LPS-induced TNF-activating factor (LITAF) and cell death involved p53 target 1 (CDIP1) can bind highly curved membranes, and mutations in the genes or their depletion result in longer tubules, and this affected the recycling of integrins and thereby cell migration [166]. Finally, the small GTPase Arf6 plays a major regulatory role in recycling from the ERC as it oversees a range of processes needed for recycling [152]. It also has a direct effect on integrin recycling [167]. For example, the Arf6 GAP ACAP1 [Arf GAP, coiled-coil (BAR domain), Ank repeat, and PH domain 1] directly binds β integrin, while the protein kinase Akt binds the α subunit, and both act as an adaptor protein for clathrin to facilitate the recycling of the integrin dimer [168, 169]. On the other hand, the Arf6 GEF ARNO/cytohesin-2 is also needed for the recycling of integrins via R-Ras signaling in tubular, EHD1-positive recycling endosomes [170].

#### Degradation

Although most integrin heterodimers are recycled, they can also be degraded. It has been shown that both α and β ligand-engaged subunits are found in MVBs. They are ubiquitinated and packaged into ILVs by the endosomal sorting complex required for transport (ESCRT) machinery, and this results in their degradation [83]. However, cancer cells have developed a way to bypass this step to increase migration on cell-derived matrix and invasion. Indeed, in cells where the expression of the oncogenic Rab25 is elevated, so is the expression of Chloride Intracellular Channel Protein 3 (CLIC3). Rab25 sorts ligand-engaged integrins into CLIC3-positive endolysosomes, and despite the degradation of the ligand, the integrin heterodimer remains active and returns to the plasma membrane. This activates Src signaling and promotes cell motility [84].

### INVs in integrin traffic and cell migration

The role of INVs in cell migration has been assessed in different cell lines in 2D and in 3D. It was found that depleting TPD54 or TPD52 by RNAi significantly reduces 2D migration on fibronectin or laminin, a phenotype that could be rescued by re-expression of wild-type TPD54, but not a mutant that cannot bind to INVs [24]. This result is important, because it is the first evidence that the presence of TPD54 protein on INVs makes them functional. Moreover, overexpression of TPD54, but not the mutant, increases cell migration, mimicking cancer conditions. Similar results were obtained in 3D migration in the invasive cell line A2780. These cells are able to invade a cell-derived fibronectin-collagen-rich matrix. Depletion of TPD52 or TPD54 greatly reduced their ability to do so [24]. These migration phenotypes were linked to integrin trafficking in two ways. First, an integrin recycling assay using TPD54-depleted cells showed a strong delay in integrin recycling. Second, integrins (α1,2,3,5,6,v and β1,5,6) were found to be cargo of INVs by proximity biotinylation and TPD54 immunoprecipitation [24]. We have seen above that the INVs are on many pathways that coincide with Rabs having an important role in cell migration and integrin trafficking, and that some of these Rabs are themselves on INVs. Specifically, INVs on the recycling pathway coupled to Rab11/Rab25, carrying integrin α5β1 are directly implicated in fibronectin-dependent migration and invasion. Other receptor types, such as growth factor receptors, can influence cell migration; it will be interesting to determine if other non-integrin cargoes are transported via INVs and if this also impact on cell motility. These results all suggest that the INVs are involved in cell migration and invasion, and that this could be the reason why TPD52 and TPD54 are linked to cancer.

## Conclusion and remaining questions

INVs were discovered by chance, during the characterization of TPD54, a protein that binds to the cytosolic face of these vesicles. INVs participate in a variety of trafficking pathways with different Rab GTPases, many of which are central to cellular migration by overseeing the recycling of integrins. Although the description of INVs is new, we do not think that the trafficking pathways that INVs participate in are novel or uncharacterized. Rather, we think that INVs are fully integrated into the membrane trafficking pathways already described; as the smallest divisible transport unit, the “atomic” vesicle. INVs are numerous, small, and highly maneuverable transport carriers. We propose that they can reach places in the cell more readily than bigger endosomes, like bike couriers in a busy city; to ensure that cargo is delivered to even the most dense regions of the cell.

Many questions remain and we highlight the four most pertinent here. First, how are INVs formed? It is possible that they are created by the vesiculation of larger carriers such as tubules. How such high curvature vesicles are formed is unclear. They do not appear to have a coat and whether or not a scission factor is required to make such uniform vesicles is an open question. Second, what is the exact role of TPD54? We know that its presence on INVs is necessary for their function [24], but its actual function is still unknown. It can bind to highly curved membranes, but whether TPD52-like proteins induce curvature or sense it, perhaps recruiting a scission factor, needs to be investigated. Third, besides the secretory and recycling pathways, what other pathways and functions require INVs? INVs have been found in association with proteins that regulate pathways for which the role of TPD52-like proteins has not yet been assessed. Autophagy and synaptic vesicle/synaptic-like microvesicle (SV/SLMV) are such pathways. They both involve vesicles of similar size to INVs (ATG9 vesicles: 30–60 nm, and SV/SLMVs: 33–42 nm) [12, 92, 115]. Moreover, proteomic analyses of both types of vesicles have revealed the presence of the TPD52-like proteins [94, 95, 117]. If the idea that INVs are simply the smallest indivisible vesicle type is correct, then ATG9 vesicles and SV/SLMVs could be thought of as flavors of this atomic vesicle type. Fourth, how do INVs move? Is their transport active, and if so, on which molecular motor do they depend and on which cytoskeletal tracks do they travel? The next few years promise to bring answers to these four questions and many more exciting developments on these fascinating vesicles.

## Data Availability

Data sharing is not applicable to this article as no datasets were generated or analyzed during the current study.
